# Self-Reported Management of Incidentally Detected Heart Murmurs in Puppies: A Survey among Veterinarians

**DOI:** 10.3390/ani14121821

**Published:** 2024-06-19

**Authors:** Marie D. B. van Staveren, Esther Muis, Viktor Szatmári

**Affiliations:** Department of Clinical Sciences, Faculty of Veterinary Medicine, Utrecht University, Yalelaan 108, 3584 CM Utrecht, The Netherlands

**Keywords:** Amplatz, auscultation, balloon valvuloplasty, catheter intervention, congenital heart disease, dogs, echocardiography, innocent murmur, patent ductus arteriosus, pulmonic stenosis, screening

## Abstract

**Simple Summary:**

Screening puppies carefully for heart murmurs during the first veterinary health visit is essential to disclose hidden congenital heart diseases. However, murmurs in puppies can also be innocent. Ideally, puppies with a possible congenital heart disease should not be sold to a new owner by their breeders. Our study aimed to investigate how easy veterinarians find differentiating innocent from pathologic murmurs, and how they manage puppies with a pathological murmur. We sent a questionnaire to veterinary practices in the Netherlands and Belgium, and analyzed 452 responses. Though 88% of the respondents find detecting a heart murmur easy, only 9% find differentiating innocent from pathologic murmurs in puppies easy. Only 80% of veterinarians recommend immediate referral of puppies with a pathologic heart murmur. Most respondents recognize that normal growth and lack of clinical signs do not rule out congenital heart disease. Though there are several centers with a veterinary cardiology specialist employed in the surveyed countries, only 43% of the respondents recommended the referral of a puppy to a veterinary cardiology specialist for murmur investigation. In conclusion, most veterinarians understand the importance of an echocardiography of puppies with loud murmurs, but they acknowledge the difficulty in distinguishing innocent from pathological murmurs.

**Abstract:**

Background: Heart murmurs in puppies can be innocent or pathologic; the latter is almost always related to a congenital heart disease. Differentiating between these murmurs can be challenging for practicing veterinarians, but this differentiation is essential to ensure the best prognosis for puppies having a congenital heart disease. Our study aimed to reveal how veterinarians manage puppies with a heart murmur. Methods: A web-based questionnaire was sent to Dutch and Belgian veterinary practices. Results: Data from 452 respondents were analyzed. Though 88% of the respondents find detecting a heart murmur easy, only 9% find differentiating innocent murmurs from pathologic murmurs in puppies easy. Of the respondents, only 80% recommend immediate additional examination when detecting a loud heart murmur during the first veterinary health check at 6 weeks of age. Most of the respondents are aware that normal growth and the absence of clinical signs do not exclude severe congenital heart disease. Of the respondents, 31% were uncertain whether early surgical intervention could lead to improved outcomes. Conclusions: Veterinarians are aware of the importance of echocardiography for puppies with a loud heart murmur, and recognize their limitations when differentiating an innocent from a pathological heart murmur in a puppy.

## 1. Introduction

Cardiac auscultation is a routine part of health checks of apparently healthy pets, which can result in the discovery of a heart murmur [1,2,3,4,5]. The first veterinary health check in puppies typically takes place at 6 weeks of age, coinciding with the first vaccination [6]. A heart murmur in a puppy might be innocent or pathologic; the latter is almost always related to a congenital heart disease [3,7,8,9]. Innocent murmurs in puppies are common as opposed to pathologic murmurs [1,2,3]. Innocent murmurs are always soft and systolic, have a short duration, the point of maximal intensity is at the region of the left or less frequently the right heart base (i.e., aortic valve), and have a musical (also described as vibratory) character [3]. The murmur characteristics of innocent murmurs in puppies are similar to Still’s vibratory murmur as it is described in children [10]. Differentiating a pathologic from an innocent heart murmur in a puppy solely through auscultation is often possible for experienced or trained observers based on the characteristics of the murmur; but this differentiation can be challenging for first-opinion veterinarians, especially in puppies with soft systolic murmurs [1,3,6,11]. However, this differentiation must be made as early as possible, ideally before the breeder sells the puppy to a new owner shortly after the first health check, for accurate prognostication and timely therapeutic intervention for the pet, prevention of emotional and financial consequences for the new owner, and reputational damage for the breeder. For two of the three most common congenital heart diseases (i.e., patent ductus arteriosus and pulmonic stenosis), effective minimal invasive catheter-based interventional therapies are available [12,13,14,15,16,17]. When intervention takes place in an asymptomatic dog (i.e., as young as possible), the prognosis is more favorable than in cases when cardiac remodeling is advanced or cardiac-related clinical signs are already present [14,16]. Last, but not least, diagnosing a congenital heart disease before the breeder sells a pup to a new owner at 8–10 weeks of age is essential for legal, financial, and emotional reasons not only for the new owner and the breeder, but also for the breeder’s veterinarian who performed the first health check [7,18].

Our study aimed to investigate how veterinarians manage puppies with an incidentally discovered heart murmur.

## 2. Materials and Methods

Our study was conducted via a web-based survey in the Netherlands and Belgium between 1 July 2021 and 15 August 2021, and it was part of a larger study [19,20]. The online questionnaire (powered by Qualtrics, Provo, UT, USA) was distributed among veterinary practices via multiple online sources: the electronic newsletter of the Royal Dutch Veterinary Association (KNMvD), a Dutch veterinary online discussion platform (Het Dierenartsen Gilde, hosted by Facebook), a mailing list of a veterinary event agency (Vitaux), newsletters of a global veterinary care franchise (Evidensia), and newsletters of two pharmaceutical companies (Boehringer Ingelheim and Vetoquinol). Veterinarians had to provide informed consent on the first page of the questionnaire before they could access the actual survey. The survey was anonymous and voluntary, but individuals were motivated to participate by the chance to win one of three complimentary entrance tickets to a Dutch veterinary cardiology symposium (Hart voor de Praktijk).

The survey consisted of 15 questions: short open questions, multiple-choice questions with an additional open answer option; questions with a scroll bar that could be set between 0 and 100, indicating a percentage or a number; and Likert scale-based questions, in which the respondents could indicate how much they agreed or disagreed with a statement on a 1 to 3 scale: agree, neutral, and disagree.

Results were exported from the online website (Qualtrics, Provo, UT, USA) into a statistical program (SPSS Statistics v28, IBM, Chicago, IL, USA). Surveys were divided into fully and partially completed categories. Data are presented as percentages. Crosstabs were used to reveal relationships. The Pearson chi-square test and Fisher’s exact test were used to test if the variables were dependent. Correlations between variables were analyzed using the Spearman correlation coefficient (r). In addition, if a relationship was suspected based on the results from the crosstabs, and there was a possibility to rank the data, this was completed to be able to test the Spearman correlation coefficient (r). The strength of correlation was classified as follows: 0.91–1.00 very strong, 0.71–0.90 strong, 0.51–0.70 moderate, 0.31–0.50 weak, and 0.01–0.30 very weak [21]. *p*-values below 0.05 were considered statistically significant.

## 3. Results

A total of 524 questionnaires were returned, of which 452 were analyzed. The remaining 72 respondents did not proceed further in the survey than the questions about the respondents’ demographics, that is why their responses were not included. Of the 452 questionnaires, 407 were fully completed and 45 were partially completed. The 45 partially completed questionnaires included besides demographic information of the respondents, responses on the ease of recognizing heart murmurs in general and the ease of differentiating innocent murmurs from pathologic ones.

### 3.1. Demography Respondents

**Figure 1:**
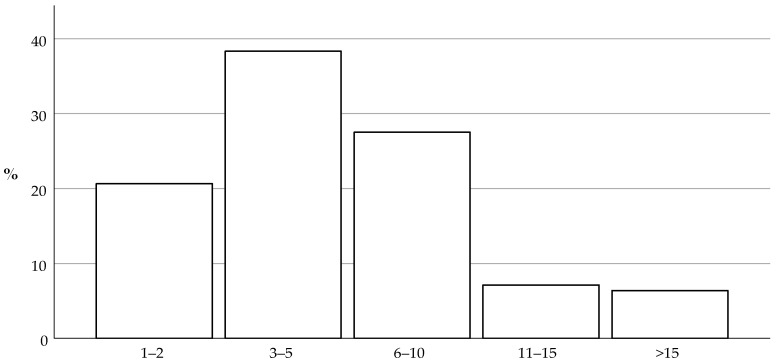
This histogram shows the size of the practice where the responding veterinarians work. The size of veterinary practice is categorized depending on the number of veterinary practitioners employed. Respondents working in a practice with 3–5 veterinarians were the most common (37%).

Of the respondents, 84% studied veterinary medicine in the Netherlands, 15% in Belgium, and 1% in South Africa, Poland, or Germany. Of the respondents, 97% practice veterinary medicine in the Netherlands; they were divided into geographic areas of conurbation (54%) and countryside (46%). The remaining 3% worked in Belgium. The number of responding veterinarians accounts for approximately 13% of practicing veterinarians focusing on companion animals in the Netherlands [22]. Respondents were categorized depending on the size of the veterinary practice, as shown in Figure 1.

Most of the respondents work solely with companion animals (82%). The remaining 18% are divided as follows: 10% work in a practice with 80–99% of their patients being companion animals, 3% work in a practice with 60–80% companion animals, 4% work in a practice with 40–60% companion animals, and 1% indicated that companion animals were the minority in their practice, between 20–40%.

Respondents were categorized following their years of experience as a veterinary practitioner: 0–5 years (29%), 6–15 years (38%), and >15 years (33%).

**Figure 2:**
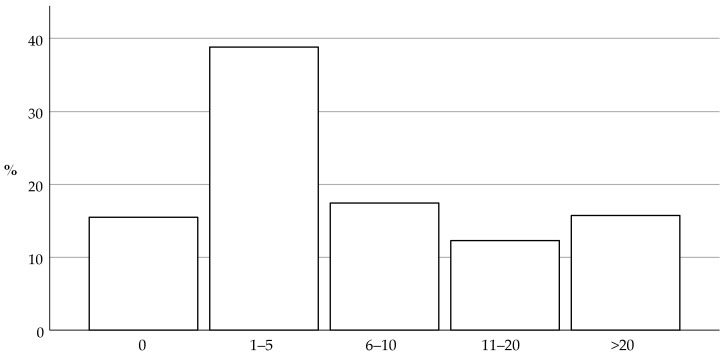
This histogram shows the estimated self-reported number of litters the respondents screen annually at the first health check at 6 weeks of age. The most frequent response was 1 to 5 litters a year.

**Figure 3:**
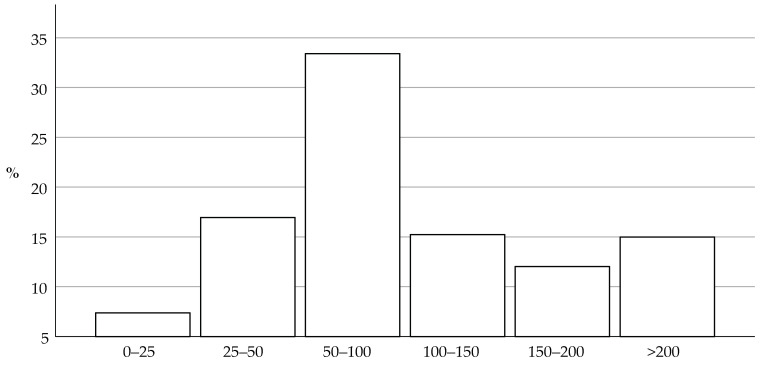
This histogram shows the estimated self-reported number of newly bought or adopted individual puppies annually that the respondents see in their practice for their second health check at around the age of 9 weeks or later. The most frequent response was 50 to 100 puppies a year.

Respondents were also categorized depending on the estimated self-reported number of litters they screen for breeders annually at 6 weeks of age, as presented in Figure 2. Finally, the respondents were categorized based on the estimated self-reported number of new individual puppies that are presented to their practice for the second vaccination and coinciding health check at 9 weeks of age or later (Figure 3).

Two-thirds of the respondents (64%) reported that they suspect a congenital heart disease in 1 to 4 puppies a year, 20% suspect this in 5 to 10 puppies a year, and 13% in more than 10 puppies a year. Three percent never suspect a congenital heart disease in a puppy.

### 3.2. Sources of Continuing Education in Cardiology

Respondents could choose multiple answers as well as write free text for this question. Various sources to stay up to date in cardiology were mentioned: 66% learn from peer-to-peer consults, 46% attend courses outside their veterinary franchise, 40% use information from representatives of the pharmaceutical industry, 35% read textbooks, 34% attend national conferences, 34% contact a veterinary cardiology specialist, 33% use online veterinary literature, 28% attend courses within their veterinary franchise, 26% use Google, 21% use peer-to-peer coaching, 16% use newsletters, and 9% attend international conferences. Four percent reported not having time for continuing education in cardiology.

The very last (47th) question of the combined survey, after the questions on adult dogs with a murmur [19,20], asked the participants about what they needed to improve the management of their cardiac cases. This question was filled in by 327 respondents. Surprisingly, none of the respondents (0%) wanted to learn more about murmurs or have training in cardiac auscultation so as to learn how to differentiate innocent from pathologic murmurs. However, 36% of them did want to get trained in echocardiography. An additional 25% said that they needed nothing else to improve the management of their cardiac cases [19,20].

### 3.3. Recognizing Cardiac Murmurs

Of the respondents, 88% find it easy to diagnose a heart murmur in dogs. Nine percent find this neither difficult nor easy, and three percent find it difficult. In contrast, only 9% find it easy to differentiate between an innocent and pathological heart murmur in a puppy, while 69% of the respondents find this difficult and 23% responded with neutral.

Of the respondents, 99% claim to document the presence of a murmur in the digital patient file of the practice (98%), in the passport (95%), or in the vaccination book (82%) of the puppy.

### 3.4. Respondents’ Knowledge of Congenital Heart Diseases

The normal growth and behavior of a puppy do not exclude a potentially serious congenital heart disease, according to 96% of the respondents. Three percent did not know if this statement was true, and two percent claimed that a serious congenital heart disease can be excluded if a puppy shows normal growth and behavior.

According to 87% of the respondents, medical or surgical intervention might be necessary for a puppy with a suspected congenital heart disease, irrespective of whether clinical signs are present. Eleven percent did not know if this was true, and three percent claimed that treatment is not necessary if a puppy has no clinical signs.

According to 62% of the respondents, the younger a puppy is when an intervention is performed for a congenital heart disease, the better the outcome is. However, 31% did not know if this was true, and 8% claimed that the outcome would not be better if the pup undergoes surgery as early as possible.

Only 80% of the respondents would advise the breeder of a puppy with a loud heart murmur to perform immediate additional examination, while 17% recommend no action but to wait. The advice of 2% would depend on the breeder’s wishes. One percent counted for miscellaneous answers such as euthanasia or serial monitoring for a couple of months before referral.

Similarly, only 83% of the respondents would advise the owner of a newly purchased puppy with a loud heart murmur detected at the second health check at the age of 9 weeks to perform immediate additional examination, typically echocardiography. At the same time, 10% of the respondents would recommend waiting instead of referral. Finally, 5% of the respondents recommend returning the puppy with a loud murmur immediately to the breeder. Two percent counted for miscellaneous answers such as tailoring their advice depending on the owner’s wishes, monitoring if a puppy would develop clinical signs, or continuing regular monitoring for a couple of months up to a year before referral.

### 3.5. To Whom Are Puppies with a Pathologic Heart Murmur Referred?

Surprisingly, only 43% of the respondents refer a puppy with a loud heart murmur for additional diagnostics to a veterinary cardiologist. The other respondents refer the puppy to either a veterinary radiologist (21%), another practice where echocardiography is performed (16%), a colleague within the same practice who performs echocardiography (10%), a mobile ultrasound service (5%), or they perform the echocardiography themselves (5%).

### 3.6. Factors Influencing the Ease of Recognizing a Pathologic Murmur in Puppies

Years of experience do not seem to influence how easily respondents recognize a heart murmur in a dog (r = 0.004, *p* = 0.931). However, less experienced (<5 years) respondents have more confidence in differentiating innocent from pathological heart murmurs in puppies than more experienced (>15 years) respondents (44% vs. 28%, r = −0.094, *p* = 0.047).

The size of the veterinary practice does not influence how easily respondents recognize a heart murmur in dogs (r = 0.061, *p* = 0.196), nor does it affect how easily they differentiate an innocent murmur from a pathological murmur in puppies (r = 0.066, *p* = 0.159). The percentage of companion animals in the respondent’s practice does not seem to influence how easily respondents differentiate an innocent murmur from a pathological murmur in puppies either (r = 0.025, *p* = 0.596).

The geographic location where respondents practice does not influence how easily they diagnose a heart murmur in dogs (*p* = 0.108), nor does it influence how easily they differentiate an innocent murmur from a pathological heart murmur in a puppy (*p* = 0.369).

How easily respondents differentiate between an innocent and a pathological murmur in puppies and the annual number of puppies a respondent sees for a first health check with the breeder (r = −0.042, *p* = 0.393), or for a second health check with the new owner (r = 0.089, *p* = 0.074), are not significantly correlated.

How easily respondents can differentiate between an innocent and a pathological murmur in puppies is not influenced by the annual number of puppies in which respondents suspect a congenital heart disease (r = 0.031, *p* = 0.535).

Veterinarians who claim to differentiate innocent from pathologic heart murmurs in puppies easily perform echocardiography themselves more often (15%), compared to the percentage of veterinarians who claim to differentiate these murmurs with difficulty, where only 4% perform echocardiography themselves (*p* = 0.04).

Veterinarians who claim to differentiate innocent from pathologic murmurs in puppies with difficulty, more often refer to a veterinary cardiologist (45%), compared to the percentage of veterinarians who claim to differentiate these murmurs easily (30% *p* = 0.04).

### 3.7. Factors Influencing the Advice to Breeders and New Owners of a Puppy with a Loud Murmur

How easily respondents differentiate between an innocent and a pathological heart murmur in a puppy does not influence their advice to a breeder of a puppy with a loud heart murmur at the age of 6 weeks (*p* = 0.810), nor their advice to a new owner of a puppy with a loud heart murmur at the age of 9 weeks (*p* = 0.467).

Years of experience do not influence the advice for a breeder of a puppy that is presented with a loud heart murmur at the age of 6 weeks either (*p* = 0.115). However, less experienced (<5 years) respondents are more likely to recommend immediate additional diagnostic examination to the owner of a puppy that is presented with a loud heart murmur at the age of 9 weeks compared to experienced (>15 years) respondents (91% vs. 62%, *p* < 0.001).

### 3.8. Factors Influencing the Knowledge of Congenital Heart Diseases

Years of experience (r = 0.026, *p* = 0.603), size of the veterinary practice (r = −0.003, *p* = 0.957), and the percentage of companion animals in the practice (r = 0.090, *p* = 0.069) do not seem to influence whether respondents think that normal growth and behavior of a puppy exclude a potentially severe congenital heart disease.

Respondents who find differentiating innocent from pathologic murmurs difficult know that normal growth and behavior can be present in puppies with a severe congenital heart disease (r = −0.159, *p* = 0.001).

Years of experience as a veterinarian (r = −0.073, *p* = 0.144) and the percentage of companion animals in the practice (r = 0.094, *p* = 0.058) do not influence whether respondents agree with the statement that medical or surgical intervention might be necessary for puppies with suspected congenital heart disease, even without any clinical signs.

Respondents from larger veterinary practices (r = 0.103, *p* = 0.038) and those who find differentiating innocent from pathologic murmurs easy (r = −0.130, *p* = 0.009) are more likely to agree with the statement that medical or surgical intervention might be necessary for puppies with a suspected congenital heart disease, even without clinical signs.

More of the less experienced (<5 years) respondents agree with the statement that the younger a puppy is when it is treated for its congenital heart disease, the better the outcome is, compared to experienced respondents (>15 years) (72% vs. 60%, r = 0.101, *p* = 0.042). Similarly, respondents of larger veterinary practices are more likely to agree with this statement (r = −0.130, *p* = 0.009). However, how easily respondents claim to differentiate between innocent and pathologic murmurs did not influence their answers to this question (r = −0.009, *p* = 0.849).

### 3.9. Influences of Sources of Continuing Education

Respondents attending courses within their veterinary franchise (95% vs. 86%, r = 0.106, *p* = 0.033), and those who attend national conferences (93% vs. 86%, r = 0.100, *p* = 0.034) find it easier to diagnose a heart murmur in dogs compared to respondents who do not attend these courses or conferences. Respondents who do not have time for continuing education find it more difficult to diagnose a heart murmur in dogs compared to those who spend time on continuing education (69% vs. 89%, r = −0.136, *p* = 0.004).

No relationship was found between attending continuing education and how easily respondents differentiate innocent from pathologic murmurs.

Respondents who use industrial representatives as a source of continuing education are more likely to agree that medical or surgical intervention might be necessary for asymptomatic puppies with a suspected congenital heart disease (91% vs. 82%, r = −0.131, *p* = 0.008).

## 4. Discussion

To manage congenital heart diseases optimally, the first step is to recognize a murmur. Luckily, most of the respondents (88%) find it easy to detect a heart murmur in dogs. However, only 9% find differentiating innocent from pathologic murmurs in puppies easy. Thus, the challenge in murmur diagnostics seems to be more in analyzing and interpreting the auscultation findings [10,23]. Based on some murmur characteristics (such as intensity, timing, and musical character), in many cases a distinction can be made by auscultation between a pathologic and innocent murmur, though this can be very challenging without sufficient training and experience [1,3,6,24,25,26,27,28]. Hence, it is not surprising that two-thirds of the respondents (69%) indicated that they find this differentiation difficult. This distinction is crucial as a puppy with a suspected pathological murmur needs to be referred for additional diagnostics as soon as possible, ideally to a veterinary cardiology specialist [1,2,10,29]. Despite the low percentage of respondents who find the differentiation of innocent from pathologic murmurs easy, none of the respondents mentioned wanting to improve their auscultation skills. Instead, many respondents wished to learn echocardiography [19,20]. Difficulties in differentiating innocent from pathologic murmurs have been reported in pediatric cardiology too, as general practitioners face the same challenges as their veterinary counterparts [28]. However, the major difference is that pediatric cardiologists get an overload of referrals of children with innocent murmurs [10,23,28]. In contrast, our previous studies show that the number of puppies with an innocent murmur that are referred to a veterinary cardiology specialist is extremely low [30].

Surprisingly, only 42% of the respondents refer a puppy with a pathologic murmur to a veterinary cardiologist. Currently, there are five EBVS^®^ (European Board of Veterinary Specialisation)-certified European Specialists in Veterinary Cardiology active in the Netherlands working in four different locations. In addition, there are centers in Belgium that are at shorter distances to owners living in the South of the Netherlands than even the Dutch centers. One explanation for this finding might be the higher consult price of a veterinary cardiologist, which could be unaffordable for some owners [31]. Because the waiting times for a consultation with a veterinary cardiology specialist in the Netherlands typically do not exceed a week, at least at the authors’ institution, long waiting times cannot be the reason for this finding. Another reason could be that the respondents judge their knowledge of congenital heart diseases sufficient to manage such patients [32]. However, according to the results of a recent study, in 88% of cases of myxomatous mitral valve degeneration veterinary cardiologists needed to alter the medical therapy after referral, while mitral valve degeneration is the most common heart disease in dogs [33]. This finding shows that optimizing therapy by veterinary cardiologists is required for even the most prevalent heart disease, for which a consensus statement is available [19,20]. Therefore, puppies with congenital heart diseases would benefit even more from referral to a veterinary cardiologist, as general veterinary practitioners seldom encounter such patients and there are no consensus statements for any of the congenital heart diseases available. Moreover, many variations in anatomy and the severity of congenital anomalies exist, and in addition a combination of various anomalies can be present, which can be challenging for even veterinary cardiology specialists and requires an individually tailored treatment recommendation. Also, given the complex nature of some congenital heart diseases, carefully approaching a pup’s medical history, clinical examination findings, and advanced echocardiographic results are pivotal for choosing the right treatment. This comprehensive approach is best carried out by a veterinary cardiologist, as it minimizes the risk of misdiagnosis and inappropriate prognostic and treatment recommendations. In addition, the cardiologists perform the therapeutic interventions themselves, knowing exactly which aspects they need to pay more attention to during the echocardiography. Therefore, veterinary cardiology specialists practically always repeat the echocardiographic examinations if these examinations were performed by a non-cardiologist before referral [30]. Good veterinary practice is considered to include referring a puppy with a potential congenital heart disease as early as possible for additional examination to the best available expert. This is also a moral obligation for practicing veterinarians, especially in a country where the highest possible resources (i.e., EBVS^®^ European Veterinary Specialists in Companion Animal Cardiology) are available within a reasonable time and distance. In our study, two-thirds of the respondents see an estimated number of 1–4 cases a year. This case load does not allow to build up experience and an up-to-date solid knowledge on the management of congenital heart diseases.

Only a soft, short-durational, systolic murmur, localized in the region of the left heart base with a musical character is typically innocent [1]. Puppies with such a murmur would not need additional diagnostics. However, while veterinary cardiologists frequently detect these murmurs, they are seldom documented by first-line practitioners [6]. This suggests a potential discrepancy in grading murmur intensity. This is particularly concerning when assessing heart murmurs in puppies and potentially missing congenital heart diseases by misclassifying a loud murmur as soft. Even loud murmurs can be missed or wrongly interpreted by practicing veterinarians [18,24,30]. Murmur intensity is a very helpful parameter as it is correlated with disease severity in patent ductus arteriosus, pulmonic and aortic stenosis in dogs [14,34,35,36].

Our study showed that less experienced respondents claim to differentiate innocent from pathologic murmurs more often than more experienced veterinarians, i.e., older respondents. A possible explanation for this outcome is the evolving emphasis on heart murmurs in puppies in the Netherlands in the past decade [6,9,18,30,37]. Also, the very first scientific publication on innocent murmurs in puppies was only published in 2015 [3]. It is plausible that veterinarians who graduated in recent years have received more training on this topic, thereby enhancing their awareness and knowledge of the characteristics of innocent and pathological murmurs in puppies, as well as the consequences of misclassified murmurs. This suggests a need for ongoing professional development to ensure that all practitioners remain up to date on current diagnostic recommendations and standards in veterinary cardiology. This, practically, means the referral of each puppy with a loud murmur to a veterinary cardiology specialist before 8 weeks of age. Another possible reason for the better self-reported performance of young respondents could be that they recognize their limitations better (by self-reflection), whereas more experienced veterinarians might rely on outdated recommendations or dogmas [38]. A study on knowledge about Lyme disease among Canadian veterinarians showed that confidence in dealing with a certain disease increases significantly with experience [39]. Surprisingly, none of the respondents indicated that continuing education on cardiac auscultation would improve the standard of care for their cardiac patients. In contrast, many of them wanted to have training in performing echocardiography. Unfortunately, the role of physical examination has become less prominent in the past decades in small animal medicine with the widespread availability of ultrasound equipment in veterinary practices. Many possible circumstances contribute to this process, but one must remember that auscultation is still the cheapest, fastest, and most practical available screening test for detecting a heart disease, and it is much easier to master than echocardiography. Recognizing innocent murmurs can be learned in a couple of hours of training by auscultation of some litters of healthy puppies under the guidance of an expert, while learning echocardiography takes a full-time 3-year cardiology residency training program and several hundreds of supervised echocardiographic examinations [6,40].

Most of the respondents urge owners to seek an immediate additional examination, i.e., echocardiography, after detecting a loud heart murmur in a puppy: 80% at 6 weeks of age and 83% at 9 weeks of age. However, 20% of the respondents still recommend no immediate action when a clearly pathologic murmur is detected at the first health check at 6 weeks of age. This is a concerning finding and an ill-advised practice. Such a practice might lead to the fact that puppies with an unrevealed congenital heart disease will be sold to a new owner. Also, not only does the early detection of a congenital heart disease provide the opportunity for timely surgical intervention, but it is also crucial for new owners and the breeders to get informed about the prognosis of the heart disease at the earliest possible stage [18,41,42]. A recent study conducted in the Netherlands showed that only 10% of dogs with a confirmed congenital heart disease were referred to a veterinary cardiologist by the breeder’s veterinarian [30]. The large discrepancy between the 80% of veterinarians who recommend immediate referral at 6 weeks of age in our survey and the 10% of referred dogs that are still owned by the breeder, as we found in a previous study, can have several causes. One reason is that, according to the findings of the present survey, more than half of dogs are not referred for echocardiography to a veterinary cardiologist, but to a veterinary radiologist or a non-specialist. Another reason could be that the survey results do not reflect the reality, because of the self-reporting bias and selection bias of the survey. The present survey was most likely completed by veterinarians who were interested in veterinary cardiology. We suspect that the daily practice of an “average” Dutch practitioner is less favorable than the results of this survey. Also, respondents tend to report in surveys the expected answer instead of their daily routine [43]. A plausible additional reason for the above-highlighted discrepancy is that breeders might not follow the veterinarian’s recommendation. Therefore, it is of utmost importance that the practicing veterinarians records the fact that a murmur was noted in an official document (such as a passport or vaccination book), which will accompany the puppy to the new owner. In our present study, almost every respondent reported that they record the presence of a murmur in the pet’s official papers. This is in contrast to our previous findings, where in one study 99% of veterinarians did not record the presence of a soft heart murmur in healthy puppies during their first health check, and in another study, only 25% of cases where a murmur was present were documented by the breeder’s veterinarian in the dog’s official papers [6,30].

In our study, most respondents recommend immediate additional examination when a puppy is presented with a moderately loud, i.e., clearly pathologic murmur. However, we do not know if this advice would be the same for a puppy with a soft systolic murmur, which could be pathologic too. Though most clinically relevant congenital heart diseases are accompanied by loud murmurs, not all are. Also, a murmur caused by subaortic stenosis becomes louder over time as peak velocity in the aorta increases with a worsening stenosis as the puppy grows [44].

Our study showed that respondents working in large veterinary practices manage potential cardiac diseases in puppies more proactively compared to small veterinary practices. Large veterinary practices often benefit from more specialized services, better access to advanced diagnostic equipment, and continuing education. In addition, patient owners who visit large practices might have higher expectations.

Puppies with most congenital heart diseases typically develop normally and show no clinical signs in the first few months to years of their lives [8,14,42,45]. Most respondents (96%) of our survey were aware that normal growth and demeanor and the absence of clinical signs do not exclude potentially serious congenital heart diseases. This is exactly why screening with cardiac auscultation at each vaccination is of utmost importance.

Respondents who admitted experiencing difficulties with differentiating innocent from pathologic murmurs tended to have actually more accurate knowledge of the surveyed aspects of congenital heart diseases. Their acknowledgment of difficulty suggests a deeper level of self-awareness and honesty about their knowledge gaps. As a result, they might actively invest in continuing education to compensate for these deficiencies, leading to a more comprehensive understanding of congenital heart diseases.

It is common sense that a puppy affected by a serious congenital heart disease could benefit from possible intervention before any cardiac remodeling and clinical signs appear. Therefore, it is noteworthy that in our study 31% of respondents were uncertain about whether early intervention could lead to improved outcomes for a puppy, while 8% even disagreed with this statement. This raises concerns because pet owners depend on their veterinarians’ advice for expert guidance, especially when their puppy might be experiencing a health issue such as a potentially serious heart condition requiring surgical intervention. For two of the most common congenital heart diseases in dogs i.e., left-to-right shunting patent ductus arteriosus and severe valvular pulmonic stenosis, catheter-based interventions have been proven to be safe and successful for decades [12,13,46,47,48,49,50,51]. A recent study on interventional closure of patent ductus arteriosus confirmed again that early intervention gave better results; among others the end-systolic left ventricular volume index was significantly lower in dogs that underwent surgery before 6 months of age [51].

The results of our study regarding sources of continuing education are in line with a previous study that found that veterinarians seldom use evidence-based medicine to enforce their decisions but rely more on peer-to-peer consults and contact with specialists [52]. Thus, most veterinarians prefer communicating with more knowledgeable veterinarians and using them as a source of information instead of searching for evidence-based information, i.e., original journal articles. Lack of time is probably an important factor in this.

Respondents in our study who reported not having time for continuing education found it challenging to diagnose a heart murmur in dogs in general. Murmurs in dogs are a common finding, and the ability to diagnose them is a crucial basic skill for any veterinary practitioner. This emphasizes that practical teaching in the curriculum and continuing education is essential, not only for the level of skills but also for veterinarians’ self-esteem. Furthermore, we found that respondents who attend courses within their veterinary franchise and national conferences claim to diagnose a heart murmur in dogs easily. The findings suggest that attending courses and conferences can positively influence skill and confidence levels. This is likely a result of the quality of education, networking opportunities, and exposure to various cases provided by these educational platforms.

## 5. Limitations

Our study depended on self-reported statements by veterinarians, which could have caused a possible bias. We did not test the actual implementation of these statements in daily veterinary practice, nor did we test the consequences of certain advice the respondents gave about puppies with suspected congenital heart diseases.

Despite all the efforts to design the questions as objectively as possible, there is a possibility that the way they were phrased influenced the answers.

As the majority (97%) of our respondents worked in the Netherlands as veterinarians, the results of the study can only say something about Dutch veterinarians.

## 6. Conclusions

Veterinarians who participated in our study diagnose heart murmurs in dogs with self-reported ease. They recognize their own limitations when differentiating an innocent murmur from a pathological heart murmur in a puppy, and they are aware of the importance of additional diagnostics for puppies with a loud heart murmur. Despite this awareness, a concerning 20% of respondents recommend no immediate action upon detecting a pathological murmur during the initial health check at 6 weeks of age. This is worrisome and calls for action to shift these veterinarians’ practices.

## Data Availability

The data presented in this study are available on reasonable request from the corresponding author.
